# More than unfamiliar environmental connection to super typhoon climatology

**DOI:** 10.1038/s41598-023-33104-3

**Published:** 2023-04-19

**Authors:** Namyoung Kang, Chan Joo Jang, James B. Elsner

**Affiliations:** 1 Department of Geography, Kyungpook National University, Daegu, 41566 South Korea; 2 Department of Oceanography, University of Science and Technology, Daejeon, 34113 South Korea; 3 Ocean Circulation Research Division, Korea Institute of Ocean Science and Technology, Busan, 49111 South Korea; 4 Department of Geography, Florida State University, Tallahassee, 32306 FL USA

**Keywords:** Climate and Earth system modelling, Climate-change impacts

## Abstract

This study employs a refined geometric variability model to look at the environmental relationship to super typhoon climatology, which is one of the major concerns about climate change and disasters. It is noted that adding only several recent years leads to a remarkable weakening of the environmental explanatory power on super typhoon climatology. Looking into the annual covariance elements, we find that the recent observations showing a group of outlying events with a particular drift are more than unfamiliar compared to the former stable relationship from 1985 through 2012. Greater uncertainty thereby amplifies concerns about the looming climate crisis.

## Introduction

The Earth’s climate is configured by air, water, ice, rocks, and living things all of which change over time^1^. If the forcings external to the climate system remain static, there is only internal variability. In the real world the climate system is stimulated by external forcings such as the Earth’s orbit, solar activity, volcanic activity, and aerosol concentrations, and so on^2^. During the recent century, increasing concentration of greenhouse gases, despite their contribution to urbanizing society, is exerting a force on the earth’s climate^3^. As a response, record-breaking extremes are continually and everywhere being reported creating a climate crisis^4,5^. The larger responses, even at the extremes, do not necessarily mean the climate is out of balance (Supplementary Fig. S2). In the study, “balance” denotes the stable relationship among the climate conditions, and potentially more dire is the possibility that an imbalance could appear as an unusual climatic response^6–8^.

Tropical cyclones (TCs) create enormous socio-economic costs^6,9–11^ but also contribute to the earth’s energy distribution^12–15^. Because of this role, the relationship between TC activity and the environment is supposed to show a certain aspect of the functional balance. This study employs a refined geometric variability model to look at the environmental relationship to super typhoon activity, which is one of the major concerns about climate change and disasters. Super typhoon climatology refers to a set of observations including total genesis frequency, rate of super typhoon occurrences, overall TC activity, and the efficiency of intensification^16^. While the climatology has shown a strong relationship with the large scale forcings of El Niño-Southern Oscillation (ENSO) and global ocean warmth^16,17^, we find more than unfamiliar connections beginning in the early 2010s.

**Figure 1:**
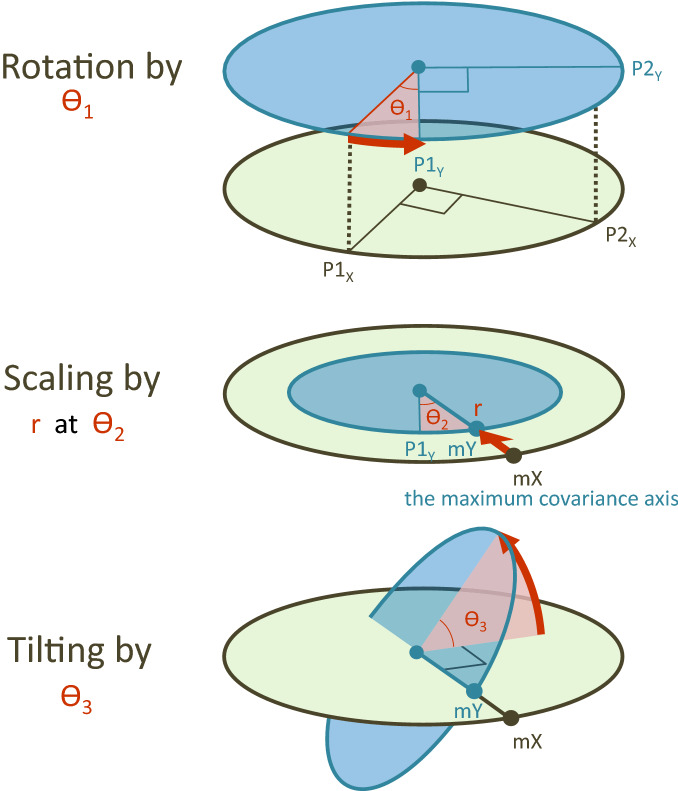
A geometric variability model. The climate connection between the two variability planes is configured by rotation ($\theta _{1}$), scaling (*r*) in the maximum covariance direction ($\theta _{2}$), and tilting ($\theta _{3}$) of the response variability plane. The geometric model gives a presentation of the response variability plane onto the explanatory variability plane in the three-dimension variability space. All variables use standardized values. The center of the planes represents the mean value, while the boundary indicates 1.0. The response variability plane and the explanatory variability plane are constructed from the TC variables and the environmental variables, respectively.

**Figure 2:**
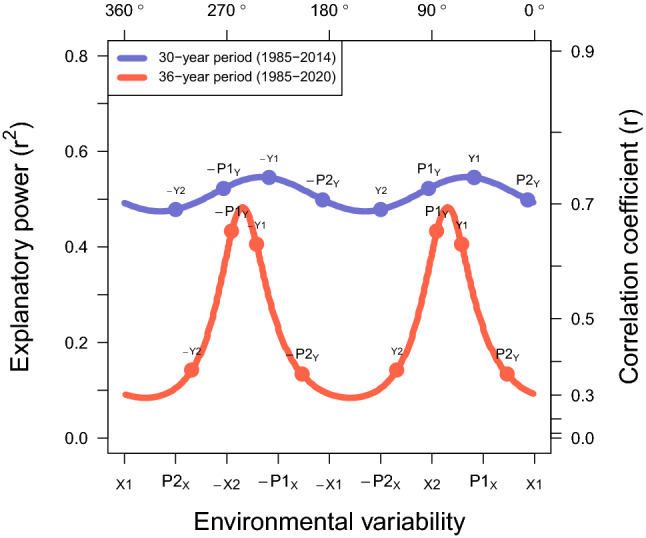
Reduction in the explanatory power of the environmental variables. Comparison of the environmental relationships to TC variables between two periods. Variables are averaged over June to November. The former 30-year (1985–2014) TC-climate connection to the environment is shown in blue, while the recent update (1985–2020) is shown in red. The positions of the TC variables show the best explanatory variability and its explanatory power. A large reduction in the explanatory power together with the changes in the corresponding directions is noted when including data from the years since 2013.

**Figure 3:**
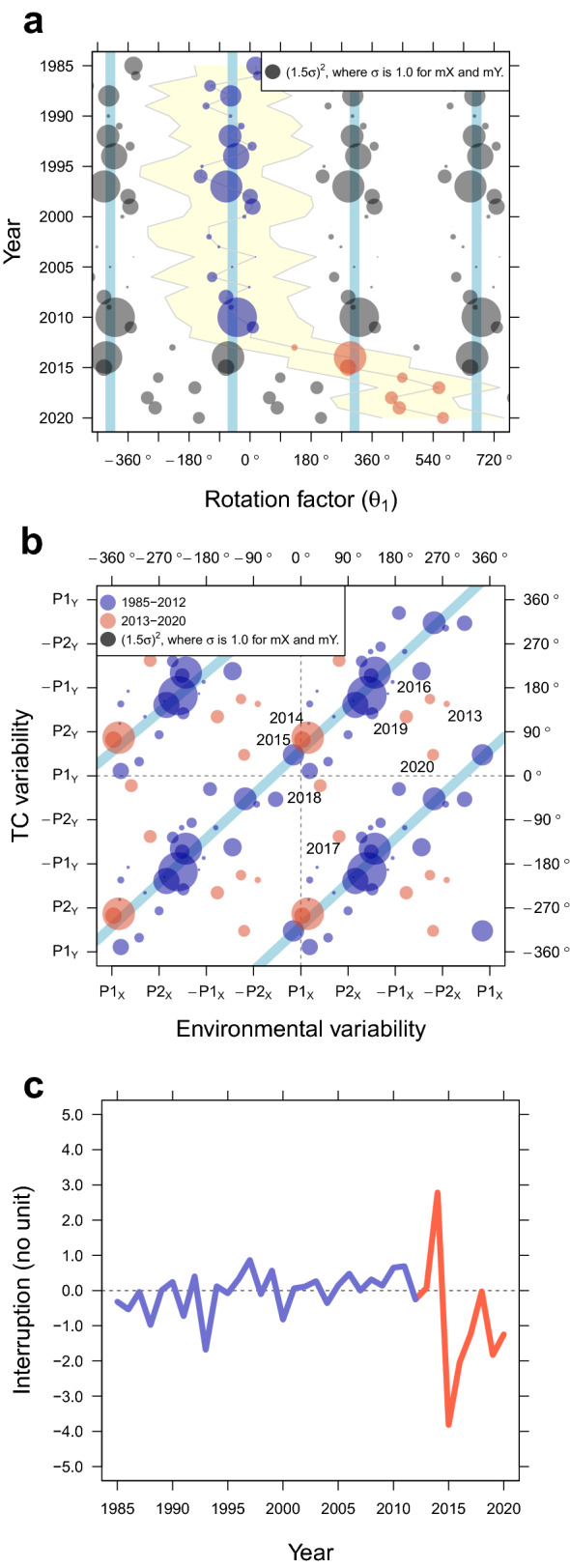
Sudden and still ongoing drift of the climate connection. The direction and magnitude of the annual maximum covariance elements ($mX_{t} \cdot mY_{t}$) distributed on (**A**) a time domain and (**B**) an XY plane, and (**C**) the interruption ($mX_{t} \cdot mY_{t} -mX_{t} \cdot mX_{t}$). All variables are annually averaged from June through November. Since the variability direction is cyclic, the distribution is tiled as a mosaic. It is noted that $\theta _{1}$ had been stable around the value of − 51.9$^{\circ }$ until 2012 (sky-blue lines), and the sudden and still ongoing drift of the climate connection began in 2013. Simultaneously, the interruption started a distinct fluctuation in 2014 (red line).

**Figure 4:**
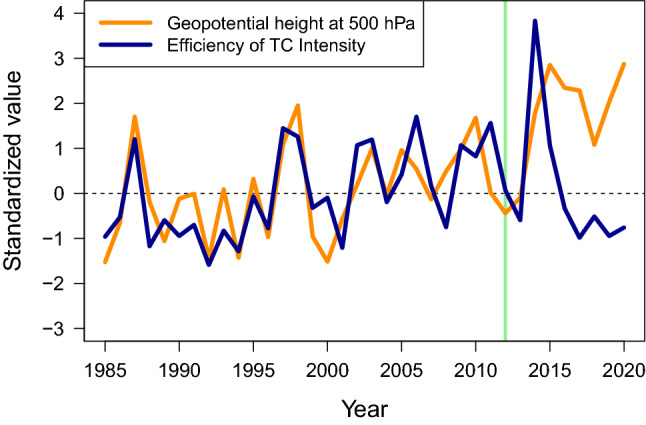
Time series of the atmospheric suppression and the efficiency of TC intensity in the western North Pacific. The atmospheric suppression is indicated by geopotential height at 500 hPa. The efficiency of TC intensity is the out-of-phase principal component of annual TC intensity and frequency. All values are annually averaged over JJASON in the tropical region of the western North Pacific (0$^\circ$–30$^\circ$N, 100$^\circ$E–180$^\circ$). Green vertical line indicates the year 2012. For demonstration, annual values are standardized by the values during the 28 years (1985–2012).

## A geometric variability model

By employing a ‘three-dimensional variability space’^17^, we refined a geometric variability model to quantify how the response variability space harmonizes with explanatory variability space and how that harmony is changing (Fig. 1). For this, the two variability planes are configured and investigated. The model includes components describing the shape of the response variability plane ($P1_{Y}$ $\sim$ $P2_{Y}$ $\sim$ − $P1_{Y}$ $\sim$ − $P2_{Y}$ $\sim$ $P1_{Y}$) juxtaposed above the components describing the explanatory variability plane ($P1_{X}$ $\sim$ $P2_{X}$ $\sim$ -$P1_{X}$ $\sim$ -$P2_{X}$ $\sim$ $P1_{X}$). The principal component analysis produces the same number of principal components as the number of input variables. Here, $P1_{Y}$ and $P2_{Y}$ are the two principal components from *Y*1 and *Y*2. $P1_{Y}$ and $P2_{Y}$ respectively show the in-phase and out-of-phase modes between *Y*1 and *Y*2. The variability space is constructed with perpendicular axes for illustration but that does not imply the variables *Y*1 and *Y*2 are orthogonal. The same for $P1_{X}$ and $P2_{X}$ from *X*1 and *X*2.

The overall shape of the response variability plane is constrained by (1) rotation ($\theta _{1}$), (2) scaling (*r*) in the maximum covariance direction ($\theta _{2}$), and (3) tilting ($\theta _{3}$) of the response variability plane. *mX* and *mY* denote each maximum covariance direction for the X-plane and Y-plane. In this variability framework, a correlation is the projection length between the variables. All variables are standardized by subtracting the mean and dividing by the standard deviation using observations over the entire period. The center and the boundary in the screen show the mean of the annual values and a correlation coefficient of one, respectively. The larger the angle between the directions defined by the variables, the weaker the correlation between them. The final projection of the response variability plane onto the explanatory variability plane shows a bird’s-eye view of how the environment is statistically connected to the TC climate, which effectively integrates all regression coefficients from individual ordinary-least-squares methods (*Y* $\sim$ *X*) (Supplementary Fig. S5).

## Two variability planes

To identify features of the TC response to environmental climate conditions, we focus on super typhoons in the western North Pacific during the period June through November (JJASON) because they are the most energetic. Considering the reliability of the best-track data^18–20^, we use observations over the period 1985–2020, inclusive (36 years). A TC has a lifetime-maximum intensity (LMI) exceeding 17 m $\hbox {s}^{-1}$. A super typhoon has an LMI of at least 65 m $\hbox {s}^{-1}$ and we use intensities estimated by the US Joint Typhoon Warning Center.

First, super typhoon climatology refers to a set of observations including total genesis frequency, rate of super typhoon occurrences, overall TC activity, and the efficiency of intensification^16^. Here, we configure a TC variability plane using the annual frequency defined by the annual number of TC occurrences and the annual TC intensity defined by the annual proportion of TCs that become super typhoons^16^ (Supplementary Fig. S6A). Other choices for annual TC intensity include the annual mean LMI and 70th percentile intensity^21^ but the number of Category 3, 4 or 5 typhoons as an inner product of the total number of TCs and the proportion of strong typhoons (i.e., the frequency and the intensity) is statistically comparable to other metrics of TC activity such as the Accumulated Cyclone Energy^22^ and the Power Dissipation Index^23^. With the intensity and the frequency denoted as *Y*1 and *Y*2, respectively, the two principal components of in-phase mode ($P1_{Y}$) and out-of-phase mode ($P2_{Y}$) are obtained. Here, the subscript “*Y*” in the variable names denotes they are the response variables to the environment. The principal components $P1_{Y}$ and $P2_{Y}$ are interpreted as the annual TC activity and the efficiency of intensification, respectively. The efficiency of intensification is the intensification at the expense of TC frequency^17,24^, and thus, the larger $P2_{Y}$ implies a larger proportion of super typhoons even with fewer TCs. The variability plane is helpful to locate and compare any available TC variables in a continuous variability space.

Similarly, we configure an environmental variability plane using the global mean sea surface temperature (GMSST) to indicate the forcing by global ocean warmth, and the Southern Oscillation Index (SOI) to indicate forcing by oceanic-atmospheric internal variability through ENSO (Supplementary Fig. S6B). These environmental variables are denoted as *X*1 and *X*2, respectively. Since a positive value of SOI reflects the La Niña pattern of ENSO, we use negative SOI as the positive sign on *X*2 to indicate ENSO variability. The environmental variability plane is defined by the two principal components of in-phase mode ($P1_{X}$) and out-of-phase mode ($P2_{X}$). The subscript “*X*” represents the explanatory variables. $P1_{X}$ indicates the in-phase variability direction of a warmer El Niño condition, while $P2_{X}$ indicates the out-of-phase variability direction of a warmer La Niña condition^17,24^. Similarly, a colder La Niña condition and a colder El Niño condition are in the opposite directions of $P1_{X}$ and $P2_{X}$, respectively. With the help of this variability space, the directions of the environmental variables are identified by their projection onto the variability plane. The *X*2 appears orthogonal to *X*1 ($r= +0.10; [-0.24, 0.41]$, 95 % confidence interval), and other variables found in the literature for describing the TC environment project onto the plane in directions similar to their counterparts.

The possibility exists that a longer-term internal variability might seem to have some trend in a shorter time window. For instance, periodical solar activity can modulate Pacific meridional mode (PMM) variability^25^, and subsequent responses could occur in the tropical SST through the wind-evaporation-SST process^26^. PMM variability could also be influenced by external factors such as greenhouse warming as well as the volcanic eruptions^27^. Regarding global warming, Niño indices such as Niño 1+2, Niño 3, Niño 3$\cdot$4, and Niño 4, as defined by tropical SSTs, cannot be free from environmental changes in the tropics^28^. Changes to the tropical SST pattern like those across the central Pacific SST indicated by the Niño 4 index can excite a PMM response through the Aleutian low variability exhibiting the Pacific decadal oscillation pattern^29^. Consequently, an environmental variable between *X*1 and *X*2 implies the merged reflection of global ocean warmth and ENSO on interannual timescales. To the extent that such a variable is associated with ocean dynamics, its response to the forcing would be slow due to thermal inertia of the ocean^30^. This would weaken the correlation between the longer-term and interannual variability. The orthogonal variability plane on interannual timescales effectively spans the environmental variability directions onto which the TC variables can be projected.

## Remarkable changes to the recent climate connection

The environmental connection to TC activity is described by associating the two corresponding variability planes. The correlation wavelets shown in Fig. 2 are understood as the expression of the directions and the lengths from this geometric projection (Supplementary Fig. S5). The thirty-year (1985–2014) connection is revisited^17^ as a prior reference (Fig. 2 blue line). We define the connection by the direction of the closest environmental variability and the explanatory power ($r^{2}$) to each TC variability. Red and blue dots in Fig. 2 show in which environmental variability direction each TC variability is best explained. Annual TC activity ($P1_{Y}$) is best explained by the variability near the El Niño variability (*X*2), while the efficiency of intensification ($P2_{Y}$) matches the variability near the global ocean warmth (*X*1) confirming how the continuous warming of the environment in conjunction with El Niño ($P1_{X}$) contributes to the continued increases of TC intensity (*Y*1)^31^. Physically, TC genesis frequency (*Y*2) in a warmer environment is hindered by active moist adiabatic warming in the troposphere^32,33^. Suppression (− *Y*2) is most effective when La Niña conditions occur during a warmer year ($P2_{X}$) which is likely to be accompanied by decreasing upward mass flux^34,35^, increasing saturation deficit^36,37^, and anomalous highs in the middle and upper troposphere^17,24^. The environmental connection to TC climate implies that the continuous warming of the environment is likely to lead to record-breaking TC intensity during El Niño as well as a falling frequency during La Niña^24^. A tight connection between the two variability planes is confirmed by the strong correlation coefficients. The explanatory power of the environment on the whole TC variability directions averages to 51 %.

The geometric model depicts how the overall TC-climate relationship changes over time. It is surprising that the TC-climate connection after adding only six more years of observations (2015–2020) is remarkably weakened in explanatory power (Fig. 2 red line). Annual TC activity ($P1_{Y}$) and intensity (*Y*1) retain some of the earlier connection, but much of the TC variability indicates weaker connections. The environmental direction of $P1_{Y}$ indicating a rotation factor ($\theta _{1}$) shows comparatively small change. Maximum covariance direction ($\theta _{2}$), the angle of the peak correlation and $P1_{Y}$, is shifted by the narrower correlation peak. Scaling factor (*r*) in $\theta _{2}$ reduced from 0.74 to 0.70 by the larger interruptions. Reduction of the correlation coefficients in most environmental variability directions implies a steeper tilting ($\theta _{3}$). $\theta _{3}$ from 46.5$^{\circ }$ to 73.1$^{\circ }$ proves that the recent climatic events were unevenly distributed over the variability plane compared to the former events. The largest change occurs in the variability direction around the response to global ocean warmth (*X*1) implying that recent additional warming produces a response that is now different (Supplementary Fig. S9). Did the past observations contribute to the appearance of a stable climate connection by chance or are the recent observations evidence of an emerging imbalance in the connection among climate conditions? Since the current correlation approach is only an average of the annual covariance elements, the correlation itself cannot answer how the climatic relationship changes over time.

## Sudden and still ongoing drift

We look into the annual covariance elements to see how the noticeable change in the geometric variability model occurred, which is considered a novel approach to validating a statistical model. Statistically, the correlation coefficient (*r*) between the two standardized variables of *X* and *Y* is calculated by $\sum _{t=1}^{N}X_{t} \cdot Y_{t}/(N-1)$, where *N* is the number of years. This study defines $X_{t} \cdot Y_{t}$ as the annual covariance element. Let’s think of an annual covariance field at certain paired variability directions (Supplementary Fig. S12). We focus on the functionality of the maximum covariance element. Because any directional covariance element ($X_{t} \cdot Y_{t}$) in year (*t*) can be functionally determined by the maximum TC variability ($mX_{t}$) and the maximum environmental variability ($mY_{t}$), the direction and the magnitude of annual maximum covariance element ($mX_{t} \cdot mY_{t}$) are the most important determinants of the TC-climate connection in a variability model. The prefixed “m” denotes annual “maximum value”.

Firstly, we examine the annual variation of the rotation factor ($\theta _{1}$). The position of $mX_{t} \cdot mY_{t}$ identifies the environmental forcing and the TC response at the same time. The angle between the variability directions of $mX_{t}$ and $mY_{t}$ implies $\theta _{1}$ which shapes overall climate relationship in a certain year (*t*). Fig. 3A shows annual variation of $\theta _{1}$ indicating how TC variability plane is positioned on the environmental variability plane. Since the variability direction is cyclic, the distribution is tiled as a mosaic. One of the key findings is that random annual $mX_{t}$ and $mY_{t}$ events are in reality constrained by a climate relationship defined by $\theta _{1}$. $\theta _{1}$ in Fig. 3B confirms that the position was stable until 2012 (around − 52°, sky-blue vertical lines), but afterwards it dramatically changed. The first notable deviation occurred in 2013, but it was not so disruptive to the former TC-climate connection because of its small covariance element (indicated by a small circle size). A line linking the nearest annual positions shows that the change persists in one direction (drifts) until recent years. On the way, 2014 and 2015 seem to come back to the former $\theta _{1}$, which makes the drift during 2014 to 2015 less pronounced. The drift of $\theta _ {1}$ resumed in 2016 and during the five-year period of 2016–2020 the observations result in a group of years around a new value of $\theta _{1}$. We cannot be sure whether this indicates a newly balanced TC-climate connection or an anomalous group of years. If this is a new balance, the former environmental connection will no longer be valid in explaining TC-climate variability.

Secondly, the annual variation of the scaling factor (*r*) in the maximum covariance direction ($\theta _{2}$) is examined. Here, the annual maximum covariance element ($mX_{t} \cdot mY_{t}$) is usefully exploited again. Though *r* cannot be estimated by a single $mX_{t} \cdot mY_{t}$, its difference from each given environmental condition ($mX_{t} \cdot mY_{t}-mX_{t} \cdot mX_{t}$) clearly shows how the environmental connection is interrupted by unknown variables (Fig. 3C). Since the horizontal dashed line at zero means no interruption, the farther values from the line imply the larger interruptions and the greater reductions in the explanatory power of the environment. Distinct unprecedented interruptions occurred in 2014 and 2015 and still fluctuate. A notable point is that, as we showed above, during 2014 and 2015 it looked as if $\theta _{1}$ temporarily returned to the former status during the drift but in reality, the TC-climate connection during these two years suffered from huge interruptions.

Lastly, the tilting factor ($\theta _{3}$) is examined by the group of recent observations. The smaller $\theta _{3}$ means the longer projection length of the TC variability onto the environmental variability plane, which occurs when the maximum covariance elements are evenly distributed over the environmental variability directions. While the points over the earlier period 1985–2012 are evenly distributed on the plot, the points over the later period are irregularly clustered with gaps indicating a large difference between the maximum and minimum covariance (see Fig. 3B) confirming a contribution to the larger $\theta _{3}$ as shown by a loss of explanatory power (Fig. 2).

The regional aspect of global warming could be looked into by the regional responses to GMSST. It has been known that the global warming indicated by GMSST significantly increases the efficiency of TC intensification by the dominant high pressure anomalies with the more unstable atmosphere in the western North Pacific^24^. Even with the drifting environmental connection to TC climatology, we find that this region has experienced the stronger environmental responses to global warming in recent years (Supplementary Fig. S11). The correlation implies that the tropical region where most TC activity occurs has become even warmer. High pressure anomaly is also shown much stronger over the same region. On the other hand, Fig. 4 shows the time series of geopotential height at 500 hPa compared to the efficiency of TC intensity which is considered to regulate TC intensity and frequency at a given level of TC activity. All values are annually averaged over JJASON in the tropical region of the western North Pacific (0$^\circ$–30$^\circ$ N, 100$^\circ$ E–180$^\circ$). The correlation during 28 years (1985–2012) drops dramatically from 0.72 {[0.48, 0.86], 95 % confidence interval (CI)} to 0.42 ([0.11, 0.66], 95 % CI) when the 36-year period (1985–2020) is applied, which implies that the recent TC intensity and frequency now cannot be effectively explained by atmospheric suppression due to global warming. The recent split of the two variables in the time series also proves that the weaker correlation does not simply mean some smaller magnitude of the environmental connection but a mixture of unexpected observations in recent years.

This analysis shows conclusively that the former environmental relationship to TC climate are far less valid, and they are drifting to a new state. The analysis is done on western North Pacific super typhoons since collectively they are the most energetic but similar changes to the TC-climate environment relationships are found for North Atlantic and eastern North Pacific hurricanes and their environments (Supplementary Fig. S13) and collectively for all basins (Supplementary Fig. S14) indicative of a global imbalance in TC climatology. This study deals with TC climatology which is only related to intensity and frequency. For a better understanding of TC climatology, the scope of future research may include TC track climatology.

## Conclusions and discussion

This study investigates the balance of the climate system by decomposing the environmental connection to super typhoon activity which is one of the central issues regarding climate change and natural disasters. A model for the environmental connection is refined using geometric factors of the TC variability plane (rotation, maximum covariance direction, scaling, tilting), which depicts the variability directions and the explanatory powers in a three-dimension variability space. The geometric variability model effectively shows the functional connection between TC climate and the environment. The framework provides a bird’s-eye view of where the individual variables are located and how they are experiencing changes on interannual timescales. It is noted that only adding several recent years leads to remarkable reduction in the explanatory power of the environmental forcings on the super typhoon climatology. This study first time shows how a deformation of the former climatic connection among variables, in a changing climate, is printed on annual covariance elements. We find that the recent observations showing a group of outlying events with a certain drift are more than unfamiliar compared to the stable connection from 1985 through 2012.

The future research could focus on how it happens. The functional imbalance may be caused by a feedback process involving TCs themselves or by changes to the larger climate conditions. On the other hand, the sudden and persistent drift suggests to us that the super typhoon climatology might be entering a “tipping point” over a threshold^38^. The environmental conditions over a tipping point may turn out to be the emergence of new environmental relationships in earth’s climate system. For example, changes to the environmental conditions induced by sea-ice loss and accelerated glacier’s melting due to global warming^39^ could be disturbing the former TC-climate connection. Increasing freshwater by the melting of Greenland’s ice sheet can weaken the thermohaline circulation^40^, and the weakened circulation would influence the SST pattern in the North Atlantic^41^. Subsequently, the modified North Atlantic SST pattern may regulate the storm activities in the tropical Pacific^42,43^, which describes a connection of the Atlantic meridional overturning circulation as a potential tipping element^44^ for super typhoon climatology. A drifting climate will affect the performance of numerical approaches as well. Despite the reliability of numerical models in simulating the future TC climate with higher resolutions^45,46^, performance is limited to the warming environment^47^ as well as to ENSO variability^48^. To the extent that the historical environmental and TC observations are used to correct and verify the models, a destabilized climate with a new TC-climate relationship to the environment will make these approaches less reliable in their predictive performance. Consequently, this study suggests that a climate-change impact could take the form of uncertain diagnostics and prognostics about TC activity and thereby magnifying concerns about how to cope with the climate crisis.

## Methods

First the response variability plane and the explanatory variability plane is constructed from the TC variables and the environmental variables, respectively. Then, the geometric association of the two variability planes is modeled in the three-dimensional space. The geometric factors are (1) rotation ($\theta _{1}$), (2) scaling (*r*) in the maximum covariance direction ($\theta _{2}$), and (3) tilting ($\theta _{3}$) of the response variability plane (see Fig. 3). The annual contribution to geometric factors and the TC-climate connection to the environment is examined by the annual maximum covariance elements.

## Supplementary Information

Supplementary Information.

## Data Availability

The TC variables are calculated from the best-track data provided by the US Joint typhoon warning center (https://www.metoc.navy.mil/jtwc/jtwc.html?best-tracks). GMATS, SOI, PMM, and PDO from the National Oceanic and Atmospheric Administration (NOAA)/Climate Prediction Center are the environmental variables. The global mean sea surface temperature (SST) is calculated from the Extended Reconstructed Sea Surface Temperature version 5 (ERSSTv5) of the NOAA/National Centers for Environmental Prediction (NCEP) reanalysis^49^. The same for the Niño indices such as Niño 1+2, Niño 3, Niño 3$\cdot$4, and Niño 4 for the following regions: (1) 10° S–0$^\circ$ and 90$^\circ$ W–80$^\circ$W for Niño 1+2, (2) 5$^\circ$ S–5$^\circ$ N and 150$^\circ$ W–90$^\circ$ W for Niño 3, (3) 5$^\circ$ S–5$^\circ$ N and 170$^\circ$ W-120$^\circ$ W for Niño 3$\cdot$4, and (4) 5$^\circ$ S–5$^\circ$ N and 160$^\circ$ E–50$^\circ$ W for for Niño 4. All SST-based variables calculated from ERSSTv5 are area-weighted values. All of the statistics and figures are made using the software R (https://www.r-project.org) and code is available online (https://rpubs.com/Namyoung/P2022a).
