# Supplementary material for: More than unfamiliar environmental connection to super typhoon climatology
Source: Sci Rep. 2023 Apr 19;13:6372. doi: 10.1038/s41598-023-33104-3 (PMC10115792; doi:10.1038/s41598-023-33104-3)
Supplement: Supplementary file 1 — Supplementary Information. [file 41598_2023_33104_MOESM1_ESM.pdf]

Supplementary Information

1 A linear perspective on the climatic balance

1. 1 Relationship among the climate conditions in a linear model

A linear model delineates the relationship among the climate conditions. Each variable in the model is considered as a proxy indicating a synthetic climate condition in association with forcing that is internal and external. Let's think of a response variable, *Y*, on the variability plane of *X*s. Supplementary Fig. 1 shows a geometric relationship among the variables. Here Y, $P1_x$, $P2_x$, *sfit*, and *E* are the standardized variables showing the standard deviation of 1.0. $P1_x$ and $P2_x$ indicate the two orthogonal variabilities, which can be formed respectively by in-phase and out-of-phase principal components from any *X* variables such as *X1* and *X2*. If we denote the best explanatory variability as *sfit, Y* can be expressed as

$$Y = \cos\varphi \cdot sfit + \sin\varphi \cdot E, \qquad (1)$$

where *E* is the residuals, and φ is the angle between *sfit* and *Y*. The first right term, $\cos\varphi \cdot sfit$ is what we mostly use for a statistical model as

$$fit = \cos\varphi \cdot \cos\lambda \cdot P1x + \cos\varphi \cdot \sin\lambda \cdot P2x. \qquad (2)$$

The second right term, $\sin\varphi \cdot E$ could be considered as the defined contribution by unknown climate conditions (meaning not observed). Though unknown, their influence could be stable as long as no change occurs to the weights on the variables.

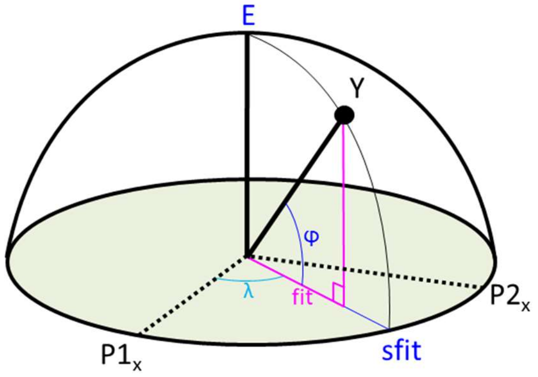

Supplementary Fig. 1. A geometric variability model of Y (single variable) on X-plane.

1. 2 Linear perspective on extreme events

Since a linear relationship already includes extreme *Y* in response to abnormal *X*, the larger responses, even at the extremes, do not necessarily mean the climate is out of balance (Supplementary Fig. 2). The balance is a matter of connection among the climate conditions, and an imbalance could appear as an unusual climatic response.

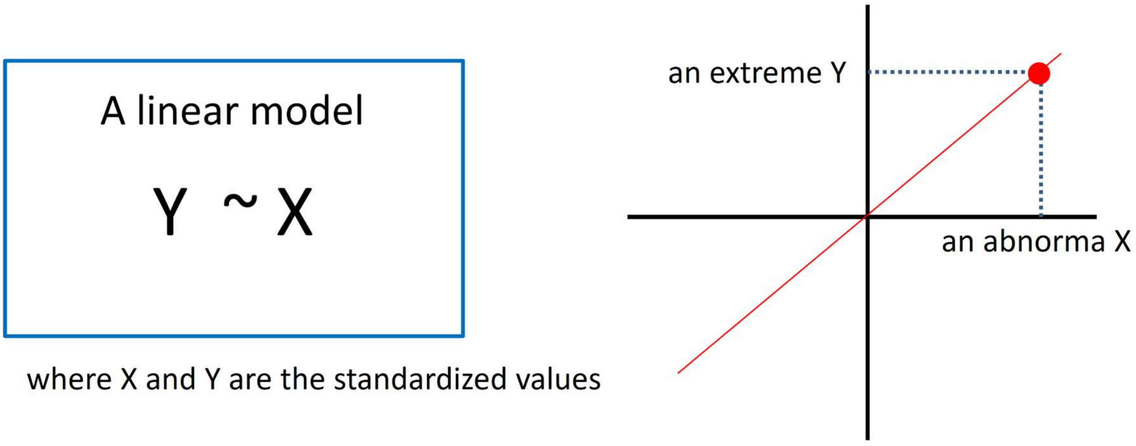

Supplementary Fig. 2. The linear perspective on extreme events.

1. 3 Verifying the geometric version of regression coefficients using R

A. Random variables of X1, X2 and Y

```
set.seed(18)
Y=scale(rnorm(10)) ; X1=scale(rnorm(10)) ; X2=scale(rnorm(10))
P1x=scale((X1+X2)/sqrt(2))[,1] ; P2x=scale((X1-X2)/sqrt(2))[,1]
```

B. Conventional linear regression coefficients

```
model=lm(Y~P1x+P2x)
coef = model$coef  #intercept and regression coefficients
a=coef[1] ; b1=coef[2] ; b2=coef[3]                # alpha, beta1, beta2
a ; b1 ; b2    #Since using standardized variables, intercept appears zero.
```

C. Geometric version of the regression coefficients

[1] Angle (lambda) **means weight**

```
fit=lm(Y~P1x+P2x)$fit
lambda=acos(cor(fit,P1x))
```

```
if(cor(fit,P2x)<0) lambda=-lambda  #negative theta for 3rd and 4th quadrants
lambda*180/pi                       #counterclockwise angle (degree) from P1x
```

[2] the best explanatory variability : sfit= w1*P1x + w2*P2x

```
w1= cos(lambda)                     #weights on A
w2= sin(lambda)                     #weights on B
w1^2+w2^2                           # confirm the sum of variance portions = 1.0
```

[3] geometric understanding of the regression coefficients : fit= β1*P1x + β2*P2x

```
r=cor(Y,fit)[,1]                    #cos(phi)
b1=r*w1                             #geometric version of beta1 = cos(phi)*cos(lambda)
b2 =r*w2                            #geometric version of beta2 = cos(phi)*sin(lambda)
b1; b2          # confirm the results are the same as those from statistical results
```

[4] Understanding of the defined contribution of the residuals : Y= B1*sfit + B2*E

```
E=scale(Y-fit)                      #Residuals by unknown variables
B1=r                                #cos(phi)
B2=sqrt(1-r^2)                      #sin(phi)
Y_geom=B1*(w1*P1x+w2*P2x) + B2*E    # the residuals are "unknown but defined" in the model
cbind(Y, Y_geom)          # confirm geometric Y
```

2 A geometric variability model for the two variability planes

2. 1 Factors constraining the shape of a geometric model

The geometric association of the two variability planes is modeled in the three-dimensional space (Fig. 1 in the main manuscript). In a geometric variability model, the shape of the response variability plane is constrained by the three factors 1) rotation (Θ_1), 2) scaling (r) in the maximum covariance direction (Θ_2), and 3) tilting (Θ_3) of the response variability plane.

ROTATION (Θ_1) indicates a counter-clockwise angle of $P1_Y$ departure from $P1_X$. Every pair of directions in the three-dimensional variability space, (i.e., X, Y), is functionally determined by Θ_1. Thus, the rotation factor represents the physical aspect of the relationship between the two variability spaces.

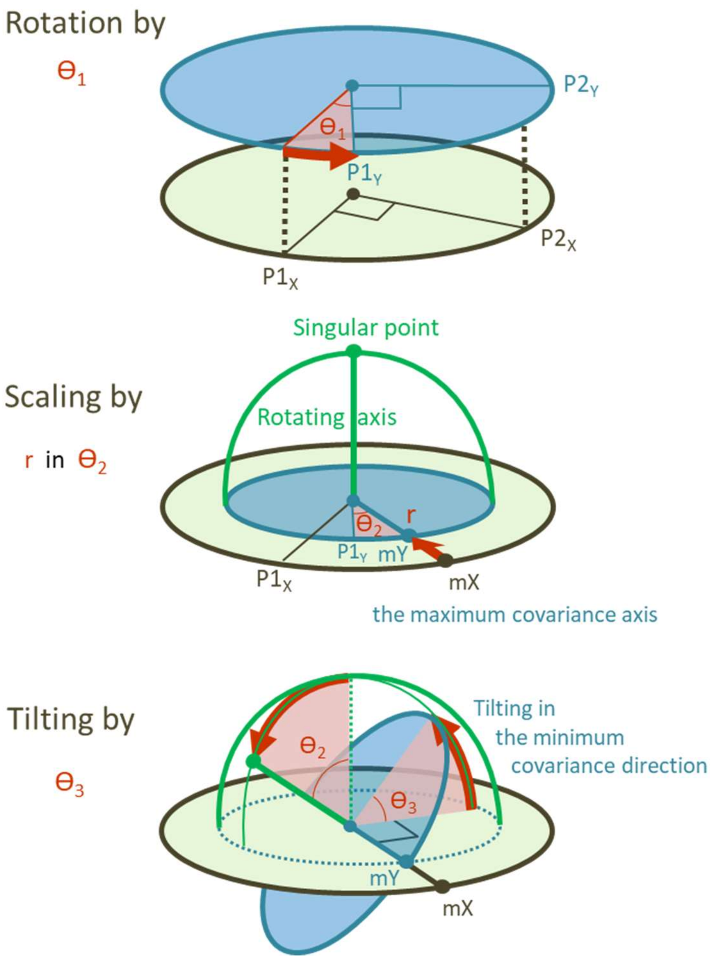

Supplementary Fig. 3. A geometric variability model for the relationship between X-plane and Y-plane.

SCALING (r) reduces the explanatory power of the explanatory variable plane in all TC variability directions. Scaling implies an interruption by undefined variability outside of the configured TC-climate relationship. The interruption is represented by the maximum correlation coefficient, r, between X and Y. Since this study defines X and Y as standardized values, the maximum correlation is understood as the maximum covariance. We denote variations having the "maximum" covariance as mX and mY, and the direction for (mX,mY).

TILTING (Θ_3) reduces the explanatory power in the orthogonal direction to the maximum covariance by definition. The smaller covariance implies lower frequency or smaller magnitude of the covariance elements in that direction. The smallest correlation can be expressed using a tilting factor (Θ_3) in the geometric variability space which makes the projection (shadow) of the response variability shorter than others.

2.2 Calculation of the rotation factor (Θ_1) and its annual inference

Variability direction of mX and mY can be expressed by the angles, δ_X and δ_Y. They are the angles from $P1_X$ and $P1_Y$, respectively. Then, the direction of the maximum covariance (Θ_2) equals δ_Y. Θ_1 can be calculated by $\delta_X - \delta_Y$ (Supplementary Fig. 4).

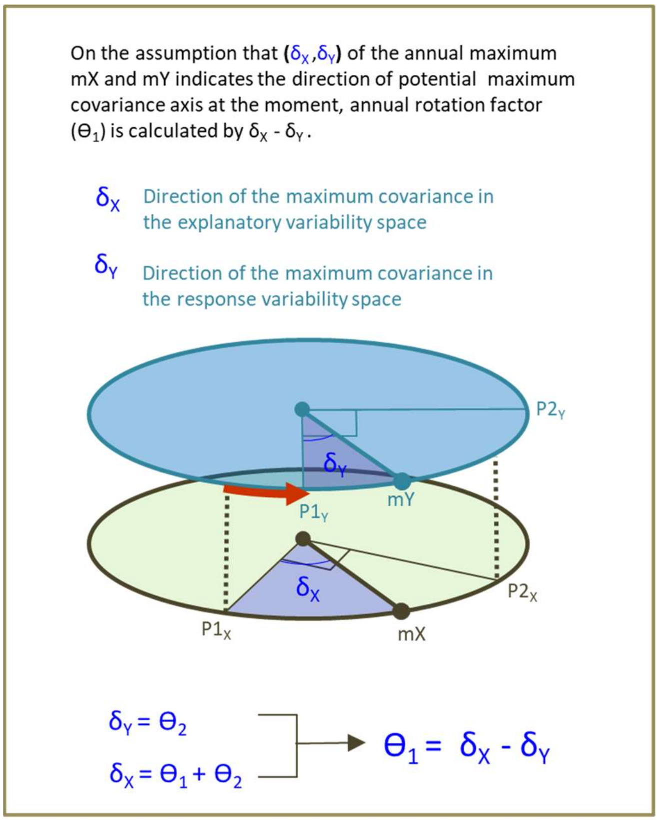

Supplementary Fig. 4. Calculation of the rotation factor (Θ_1).

Using annual observations, temporal variation of Θ_I can also be investigated. Annual Θ_I can be calculated by $(\delta_X - \delta_Y)_t$, which is difference between δ_X and δ_Y from the annual maximum covariance elements $(mX,mY)_t$ in a certain year (t).

2.3 Bird's-eye view of the climate connection

Climate connection can be expressed by the projection lengths of a response variability plane on the explanatory variability plane. Climate connection between the two variability spaces can be understood as the directional covariances, which shows a bird's-eye view of the climate connection. Projection length at each direction of paired *X*-*Y* variabilities represents the correlation which is the square root of the explanatory power of the explanatory variability.

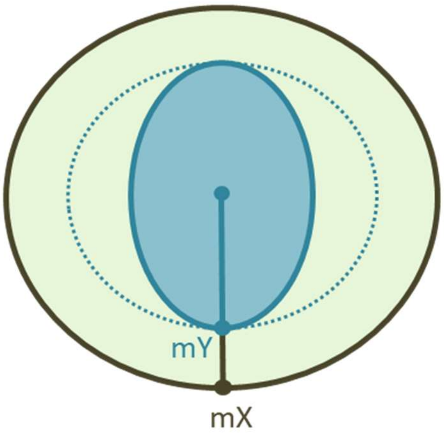

Supplementary Fig. 5. Bird's-eye view of the climate connection.

3. Two variability planes

3.1 Two variability planes in a three dimension variability space

Two variability planes are shown in Supplementary Fig. 6. All variables are annually averaged over June to November for the 36-year period of 1985 – 2020. A TC variability plane is configured by the two orthogonal variables, $P1_Y$ and $P2_Y$, which are the principal components of intensity (*Y1*) and frequency (*Y2*). Annual quantile at 0.7 probability level (*q0.7*), annual number of category 3 to 5 TCs (*C345*), its proportion in the total (*C345p*), *ACE*, and *PDI* are also shown. For the environmental variability plane the variables are global ocean warmth (*X1*) and El Niño (*X2*) and the principal components are $P1_X$ and $P2_X$. Global air temperature at surface (*GMATS*), and the regional SST indices such as Niño 1+2 (*NINO12*), Niño 3 (*NINO3*), Niño 3·4 (*NINO34*), and Niño 4 (*NINO4*) are also shown. The Pacific meridional mode (*PMM*) and the Pacific decadal oscillation (*PDO*) are also shown. The variation of *X1* is removed from *X2* (*remWarm*) to uncover the variability orthogonal to global warming. Outer and inner circles indicate the correlation coefficients of 1.0 and .5, respectively.

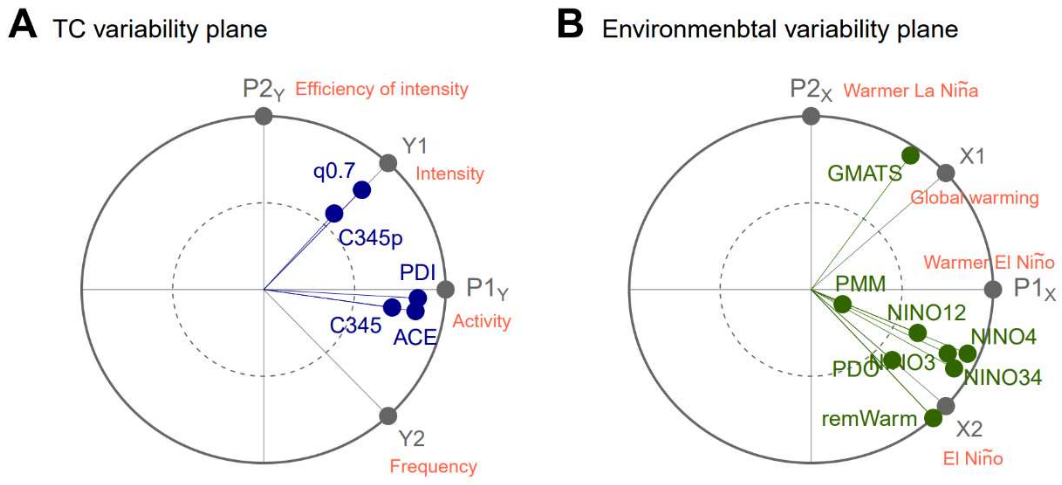

Supplementary Fig. 6. Two variability planes for (**A**) TC variables and (**B**) environmental variables. All variables are annually averaged over June to November for the 36-year period of 1985 – 2020. Each projection length indicates the largest correlation coefficient in the environmental direction.

Supplementary Fig. 7 shows that both variability planes are comparably stable over time. The positions of the gray circles represent the indices during the former period (1985 – 2014) compared to the colored circles for the total period (1985 – 2020). It shows comparably small changes in the directions of the variability indices. This is to support that each variability plane mostly shows a stability at its own. On the other hand, it is noted that some indices such as PMM and PDO change features.

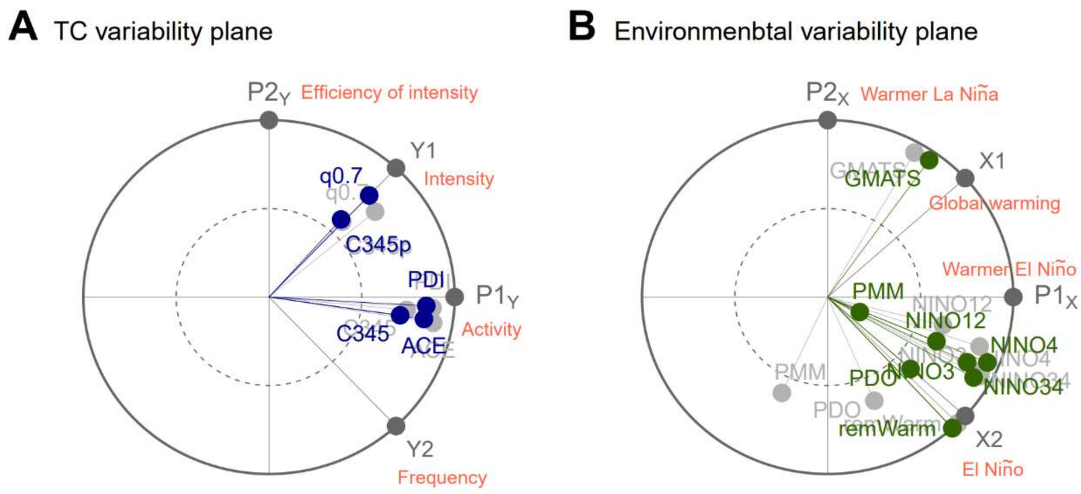

Supplementary Fig. 7. Comparison of the variability directions in Supplementary Fig. 6 with those for the former period (1985 – 2014, gray circles).

3.2 Intensity index using super typhoon activity

Supplementary Fig. 7 shows that both variability planes are comparably stable over time. Annual TC intensity could be expressed in various ways. Annual TC intensity can be indicated by the annual probability level of a certain LMI (lifetime-maximum intensity) threshold. For example, when 130 kt is taken for an LMI threshold, the TC intensity is understood as the annual proportion of super typhoons. Here, 130 kt is used as a fixed quantile for the variable probability level. On the other hand, an Inverse relationship between the probability level and quantile provides the same information, in principle (Supplementary information of Kang et al. (2019)). That is to say, from a functional perspective, the annual quantiles at a fixed probability should have the same annual variation. The relationship is similar to isobars in the weather chart, which provides pressure values at a fixed height but those are also understood as the heights at a fixed pressure level. The probability level can be

chosen subjectively. If the quantiles at 0.5 probability level are used the TC intensity is indicated by the annual medians. If a study intends to define TC intensity using stronger events, higher levels such as 0.7 can be used.

In this study, *Intensity* is indicated by the proportion of super typhoons among the total number of TCs. Since the total number is indexed by *Frequency*, the number of super typhoons equals to Intensity $\times$ $Frequency$. The difference between Intensity and conventional TC activity indices such as ACE and PDI lies in that ACE and PDI depend on frequency, duration as well as intensity.

Reference

Kang, N., D. Kim, and J. B. Elsner, The contribution of super typhoons to tropical cyclone activity in response to ENSO, *Scientific Reports*, 9, 9046 (2019).

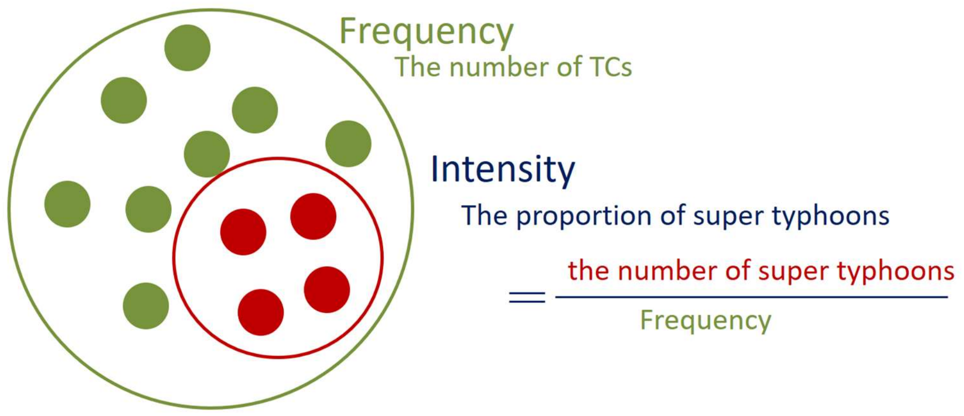

Supplementary Fig. 8. Indices of intensity and frequency forming a TC variability plane.

4. Changes to the geometric factors in the variability model

4.1 Correlation coefficients for all variability directions

Supplementary Fig. 9 shows the correlation coefficients for all variability directions. The left panel shows the results during the 30 years (1985 – 2014) and the middle during the 36 years (1985 – 2020) which provides the most recent statistics. Each squared maximum correlation (r^2) on TC variabilities is considered to be the explanatory power of the associated environmental variability as shown in the main manuscript (see Fig. 2). The right panel shows the difference in the correlation coefficients. The decrease in the positive correlations is the mirror of the increase in the negative correlations as the variability direction is cyclic. The anomalies finally result in the smaller r^2 than the former 30 years. While it shows overall changes, the largest change is found around the response to the global ocean warmth (*X1*), which implies the current change might be closely related to unfamiliar environmental conditions induced by global warming.

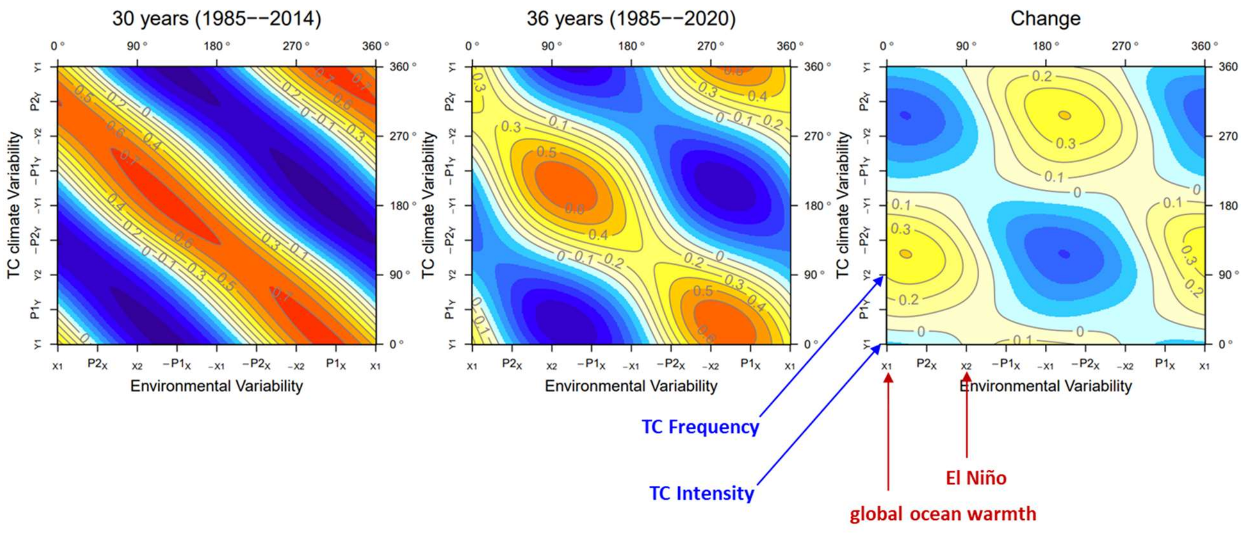

Supplementary Fig. 9. Correlation coefficients for all variability directions.

4.2 Changes to the correlation maps

Based on the findings in Fig. 3, we now examine the difference of the correlation maps between the 28-years (1985 – 2012) and the 36 years (1985 – 2020). Supplementary Fig. 10 shows the correlation of GMSST (i.e., interannual variation of global ocean warmth) with regional SST and geopotential height at 500 hPa (GHT500). To find clearer results, ENSO (indicated by SOI) is removed from the variables at all grid points before the correlation coefficients are computed. When compared to 28-year (1985 – 2012) observations, 36-year (1985 – 2020) results show that the pattern of regional responses is consistent over time, and at the same time the correlations with global warmth has become distinctly stronger in the western North Pacific. Considering that the larger GHT500 in the past observation has given more suppression on TC activity, we may expect the larger TC intensification at the expense of TC frequency. However, Fig. 4 in the main manuscript reveals a different aspect from our inference on the TC climatology.

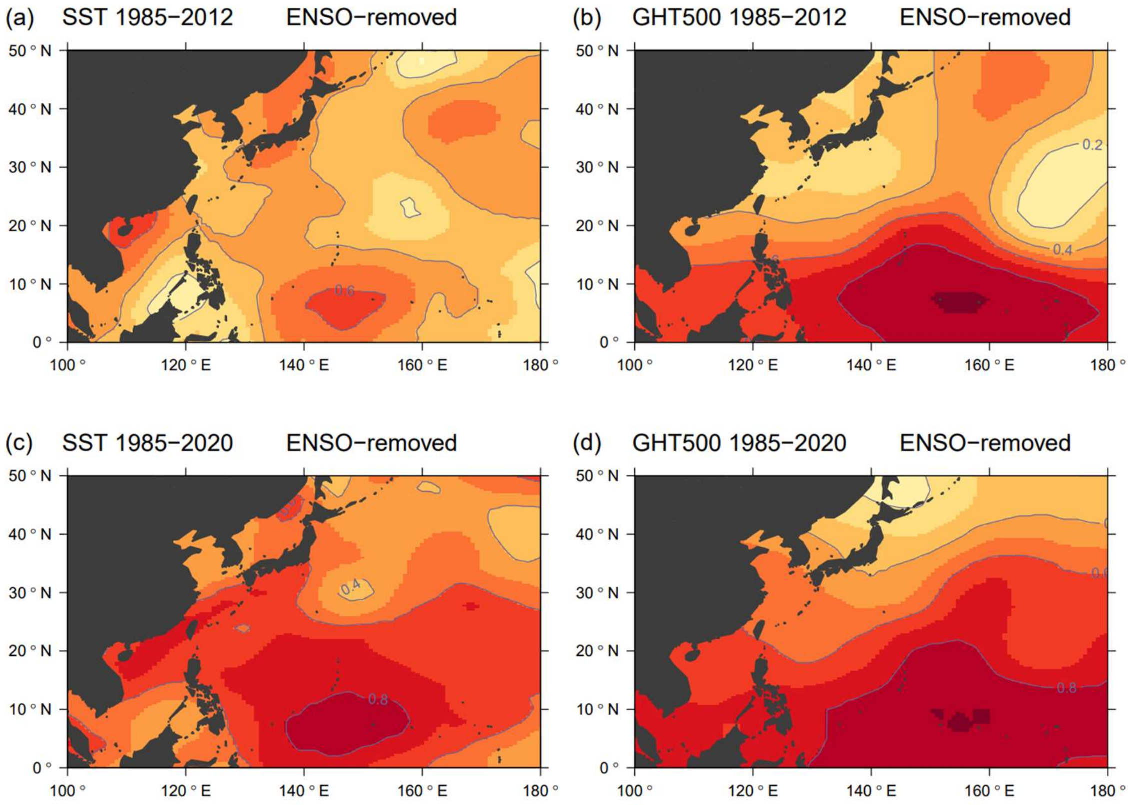

Supplementary Fig. 10. Correlation map of GMSST with (a, c) regional SST and (b, d) geopotential height at 500 hPa for the periods of 28 years (1985 – 2012) and 36 years (1985 – 2020), respectively. ENSO (indicated by SOI) is removed from the variables at all grid points before the correlation coefficients are computed.

5 Annual covariance element

5.1 Correlation coefficient and its annual covariance element

A correlation coefficient (r) between the two standardized variables of X and Y is calculated as $\sum_{t=1}^{N} Xt \cdot Yt \, /(N-1)$, where N is the total number of years, i.e., 36 (years). This study defines $X_t \cdot Y_t$ as the annual covariance element. The mathematical expression of r could be rewritten as the $\sum_{t=1}^{N} [Xt \cdot Yt \, /N] \cdot [N/(N-1)]$, showing the average of the annual covariance element multiplied by the vessel's correction (Supplementary Fig. 10).

Average of $X_t.Y_t$ Vessel's correction

$$r = \frac{\sum_{t=1}^{N} X_t.Y_t}{N} \times \frac{N}{N - 1}$$

Supplementary Fig. 11. Correlation coefficient and its annual covariance element.

5.2 Correlation coefficient and its annual covariance element

The following are the steps through which Fig. 2 (main manuscript) is made. Here, the steps are explained in reverse order (Supplementary Fig. 11).

6) Fig. 2 (main manuscript) delineates the projection lengths by how the geometric variability model is shaped (see Supplementary Fig. 5).

5) Blue and red lines in Fig. 2 (main manuscript) are the projection lengths from a geometric variability model. The geometric model effectively demonstrates the entire correlation field using only the four geometric factors (Θ_1 , Θ_2, r, Θ_3).

4) Lines in Fig. 2 (main manuscript) are the correlation ridges in a correlation field defined by X-plane and Y-plane. Any point indicated by an X variable and an Y variable produces a correlation (r) by $\sum_{t=1}^{N} Xt \cdot Yt$ / (N-1), and the largest correlation at the ridge is detected among all correlations at each TC variable.

3) As the correlation field is sort of an average of annual covariance fields. This study defines the annual covariance element as $X_t \cdot Y_t$. Among all annual covariance elements, this study denotes the annual maximum covariance element as $mX_t \cdot mY_t$. The prefixed "m" denotes annual "maximum value".

2) $mX_t \cdot mY_t$ implies how the two variability planes are combined in a certain year (t). This is what the current study focuses on. In the main manuscript, Fig. 3 describes how the annual features led to the dramatic change in Fig. 2 (main manuscript).

1) All X variables and Y variables are functionally determined by the projection of mX_t and mY_t, respectively.

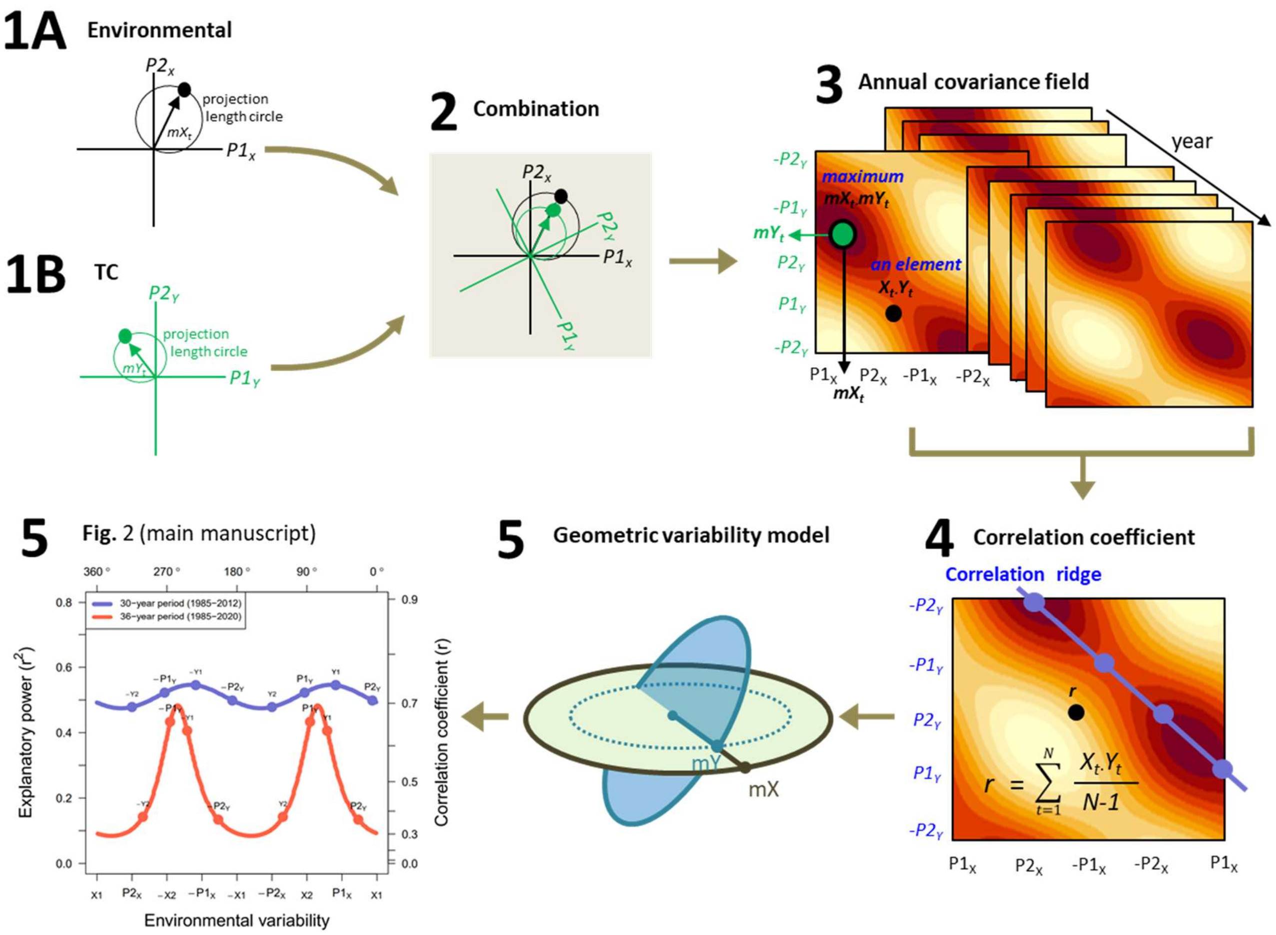

Supplementary Fig. 12. Conceptual diagram depicting the role of annual maximum covariance ($mX_t \cdot mY_t$) producing the correlation coefficient.

6. Drifting climate connection observed in other basins

Supplementary Fig. 12 shows the results from JJASON data in the eastern North Pacific and the North Atlantic, which observe a sufficient number of super typhoons for reliable statistical analysis. Big reductions of the explanatory power are apparent in these regions, too. The drifting climate connection in recent observations is also clear in both regions. Though the last observation may look like a return to the former Θ_1, the recent trend of a drifting climate connection makes this seem likely to not stop anytime soon. Observed Θ_1 in recent several years for both ocean basins suggests the former climate functionality may not work properly. A possible imbalance of the climate system is also supported by consistently fluctuating interruptions around zero line (given environmental conditions).

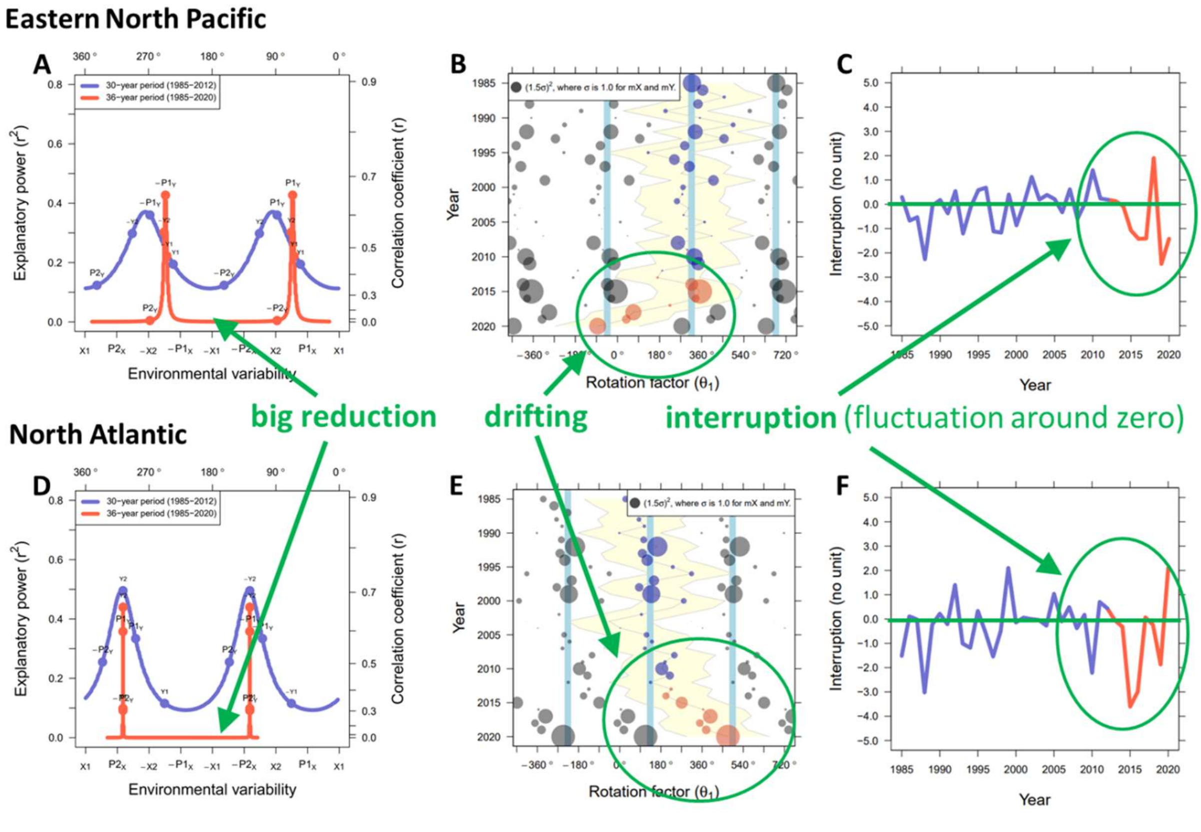

Supplementary Fig. 13. Drifting climate connection observed in the eastern North Pacific (**A**, **B**, **C**), and the North Atlantic (**D**, **E**, **F**). The best-track data from the US National Hurricane Center are used for the same period as Fig. 2 and 3 in the main manuscript.

Global analysis is done by applying twelve-month (Jan. to Dec.) observations, which shows a clear reduction in the explanatory power, too (Supplementary Fig. 13). While the last few years may look as if the climate connection came back to the former Θ_1, the drifting of the climate connection is apparently seen to have a certain trend. Interruptions are also clear to see the climate system is suffering from unfamiliar conditions on a global scale.

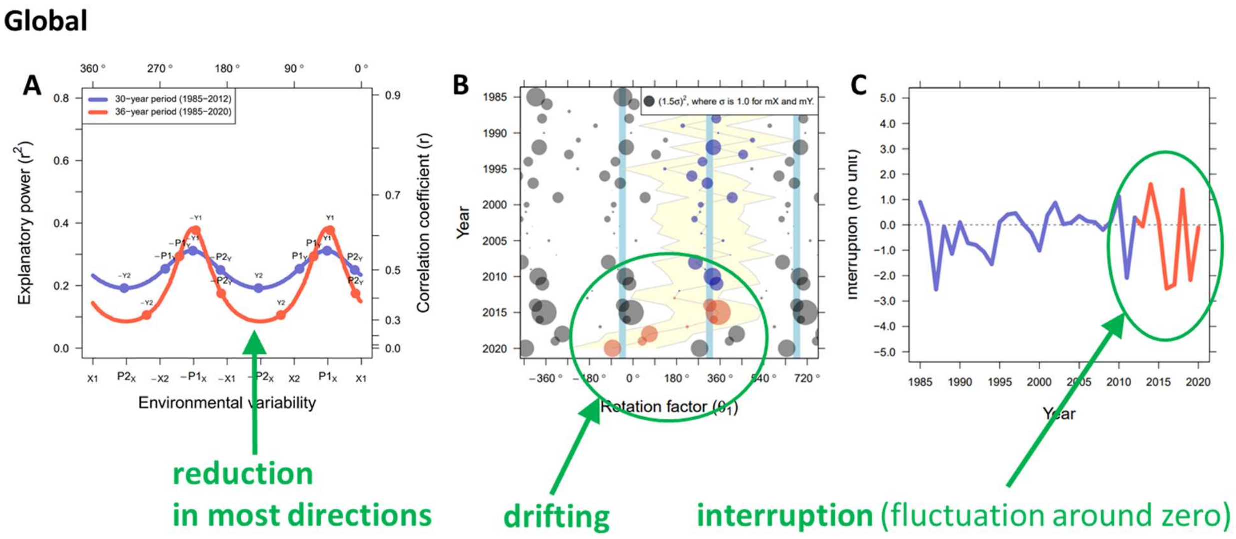

Supplementary Fig. 14. Same as in Supplementary Fig. 12, but for global super typhoons. For analysis, a global best-track data set is prepared by the observations from JTWC and NHC which consistently provide 1-min wind speeds over the regions.
